# Improving Severity Scoring of Food-Induced Allergic Reactions: A Global “Best-Worst Scaling” Exercise

**DOI:** 10.1016/j.jaip.2021.06.056

**Published:** 2021-11

**Authors:** Aisling Stafford, Joan Bartra, Antony Aston, E. N. Clare Mills, Montserrat Fernandez-Rivas, Paul J. Turner

**Affiliations:** a National Heart and Lung Institute, Imperial College London, London, United Kingdom; b Hospital Clínic de Barcelona, Barcelona, Spain; c Department of Paediatric Allergy, The Royal London Hospital–Barts Health NHS Trust, London, United Kingdom; d Division of Infection, Immunity, and Respiratory Medicine, Manchester Institute of Biotechnology, University of Manchester, Manchester, United Kingdom; e Servicio de Alergia, Hospital Clınico San Carlos, IdISSC, Madrid, Spain

**Keywords:** Anaphylaxis, Education, Food allergy, Severity score, ASCIA, Australasian Society of Clinical Immunology and Allergy, BSACI, British Society for Allergy and Clinical Immunology, BWS, Best-worst scaling, EAACI, European Academy of Allergy and Clinical Immunology, NIAID/FAAN, National Institute of Allergy and Infectious Disease and the Food Allergy and Anaphylaxis Network, SEIAC, Spanish Society of Allergy and Clinical Immunology, WAO, World Allergy Organization

## Abstract

**Background:**

There is no current consensus on assigning severity to food-induced allergic reactions, for example, to assess the efficacy of allergen immunotherapy. Existing severity scores lack the capability to discriminate between non-anaphylaxis reactions of different severities. Attempts are ongoing to develop a more discriminatory score, which should ideally be data-driven and validated in multiple cohorts.

**Objective:**

To undertake an exercise using best-worst scaling (BWS) to define a potential gold standard against which severity scoring of food-induced allergic reactions can be refined.

**Methods:**

We undertook a global survey to better understand how health care professionals rate the severity of food-induced allergic reactions, using BWS methodology. Respondents were given a number of patient case vignettes describing real-world allergic reactions and asked to select the pair that, in their opinion, reflected the maximum difference in severity. Responses were then modeled and a preference score (representing severity) determined for each scenario. Scenarios were also scored using existing published scoring systems and the scores compared with the BWS score using Spearman r correlation and Cohen kappa. Given the differences in definitions of anaphylaxis globally, we also evaluated differences in BWS ranking depending on the geographical location of respondents.

**Results:**

We received 334 complete responses, 183 (55%) from Europe and 65 (20%) from North America. Perception of severity of some reactions appeared to be affected by geographical location. The comparison of BWS ranking with current grading systems identified significant issues that varied from one grading system to another, such as prominence to some symptoms (eg, vomiting) that skew grading when using scoring systems not designed for food allergy. In general, current scoring systems poorly discriminate against more mild symptoms and often overestimate their severity.

**Conclusions:**

These data provide a methodology free of user scale bias to help define a potential, consensus-driven gold standard that can be used to guide and validate the development of improved grading systems to score food-induced allergic symptoms and highlight areas for education where there is the potential to miscategorize severity.

***What is already known about this topic?*** Existing severity scores used to grade food-induced allergic reactions have significant limitations, including an inability to discriminate between non-anaphylaxis reactions of different severities.

***What does this article add to our knowledge?*** Using a novel, consensus-based approach to eliminate user scale bias, we report a best-worst scaling exercise to help define a potential, consensus-driven gold standard to improve existing severity scores for food-induced allergic reactions.

***How does this study impact current management guidelines?*** This study identified specific limitations with existing severity scores as well as provided a potential method, free of user scale bias, toward defining a consensus-driven gold standard that can be used to guide and validate future severity scores.

## Introduction

Immunoglobulin E–mediated allergic reactions form a spectrum of severity, from very mild self-limiting subjective symptoms to potentially life-threatening anaphylaxis.^1^ Severity is a complex, multidimensional construct that is influenced by exposure dose and route as well as by cofactors such as age, comorbidity, and exercise.^2^ There is significant heterogeneity in symptoms between different food allergens, different individuals, and even between episodes within the same individual caused by the same trigger.^2^

For food allergy, severity assignment is important. For patients, their caregivers, and healthcare professionals, severity assignment is used to diagnose anaphylaxis and, thus, the indication for epinephrine. The increasing acceptance of allergen immunotherapy as a treatment modality for food allergy has highlighted the need for a validated symptom grading system to assess the impact of treatment on symptoms following allergen exposure (and not just an effect on allergen thresholds—the dose needed to trigger allergic symptoms). As research into food allergy and its treatment increases, the need for consistency in recording results is vital for analysis, interpretation, and service evaluation. There is also a need to define severity in terms of allergen risk management within the food industry and for regulatory authorities such as the U.S. Food and Drug Administration.

However, the assignment of severity can be difficult to capture, not only due to differences in anaphylaxis criteria used internationally but also the perception of reaction severity among different stakeholders.^2^, ^3^, ^4^ Perception of severity may be influenced by experience and recognition of symptoms. Patients or their caregivers may under- or overestimate severity: for example, parents may perceive significant but isolated skin signs such as facial angioedema as life-threatening, whereas experienced clinicians recognize that this is a common presentation of food-allergic reactions in young children. In contrast, some parents may attribute wheeze to a viral illness (particularly in a child prone to recurrent episodes of viral wheeze) and fail to recognize that this may imply anaphylaxis if occurring following allergen consumption.^2^ Perception of severity may be skewed for the food industry and regulatory bodies—but for different reasons. In the commercial sector, the perception of severity can be driven by brand protection and reputational issues. A clinically mild reaction may have significant ramifications (such as a food recall) if, for example, the reaction results in an unintended medical encounter.^2^

A number of scoring systems are reported in the literature, all of which have been applied to food-induced allergic reactions.^5^, ^6^, ^7^, ^8^, ^9^, ^10^, ^11^, ^12^, ^13^, ^14^, ^15^, ^16^, ^17^, ^18^, ^19^ However, only some were intended for this purpose^5^, ^6^, ^7^^,^^12^; others were developed to assign severity for non-food–induced allergic reactions and have been used beyond their initial intent. For example, the Ring and Messmer grading^13^ was originally designed to assess colloid-induced infusion reactions, whereas those by Mueller^10^ and Lockey et al^14^ were developed for venom allergy. Their application to food-induced reactions may assign a greater level of severity, for example, owing to the relative importance of vomiting as an indicator of severity in drug- and venom-induced reactions, compared with food-induced reactions in which vomiting is more likely to represent a local rather than a systemic response to allergen.

**Figure 1:**
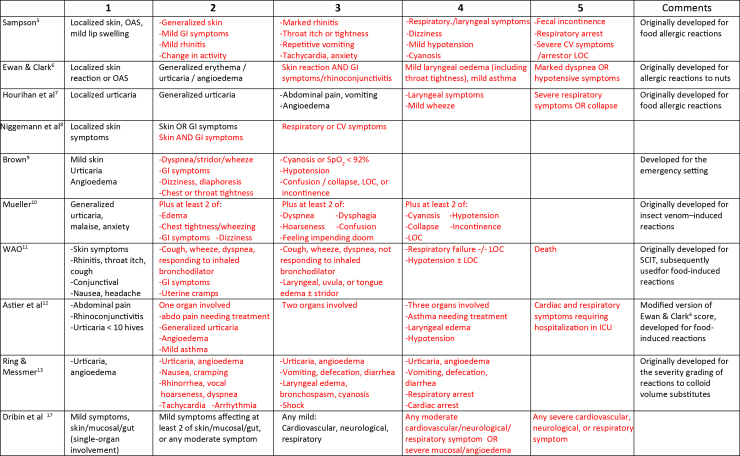
Representation of a selection of existing severity grading systems used for food allergy. Red text indicates symptoms which could be consistent with anaphylaxis as defined by the NIAID/FAAN criteria. *CV,* Cardiovascular; *GI,* gastrointestinal; *ICU,* intensive care unit; *LOC,* loss of consciousness; *OAS,* oral allergy symptoms; *SCIT,* subcutaneous allergen immunotherapy; *SpO*_*2*_*,* pulse oximetry.

Existing severity scores cover a spectrum of clinical presentations with a wide range of organ-specific outcomes, making interpretation and translatability challenging. Most operate on an ordinal scale, in which non-anaphylaxis symptoms are limited to just 1 or 2 severity grades (Figure 1), despite such reactions constituting the majority of allergic events in real life. Recent expert panels have attempted to address these limitations and develop an improved severity score.^3^^,^^4^^,^^17^ A standardized approach would certainly be useful in assessing the efficacy of interventions and facilitate allergen risk management. A universal severity score should ideally be data-driven and validated in multiple cohorts.^3^^,^^20^ This requires defining a gold standard against which such a score can be compared. In this study, we undertook a global consensus exercise to develop a methodology that might define such a reference, which could then be used by other groups to facilitate the development of a data-driven severity score.

## Methods

**Figure 2:**
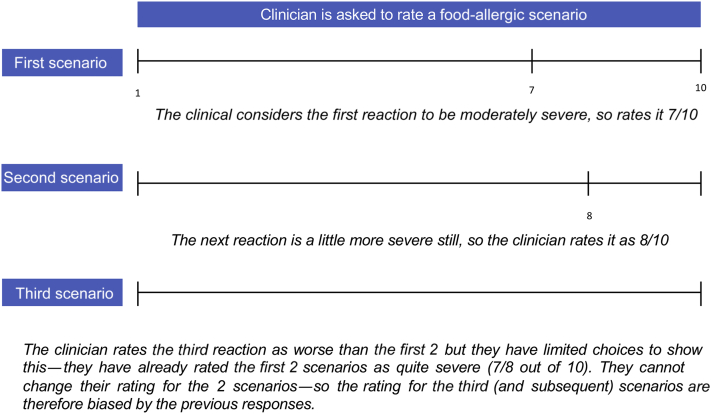
User scale bias.

We undertook a global survey of healthcare professionals affiliated to specialist allergy societies to rate the severity of different food-induced allergic reactions, using best-worst scaling (BWS).^21^^,^^22^ This methodology, also referred to as MaxDiff, is an efficient method of data collection (frequently used in market research) that uses a statistical trade-off technique that represents a model of choice process that eliminates user scale bias (Figure 2).

**Figure 3:**
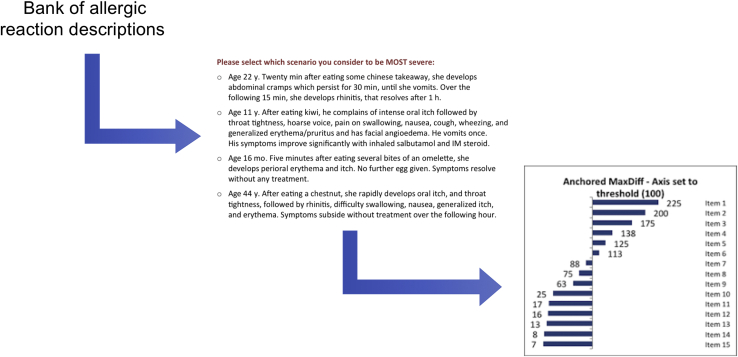
BWS methodology. *IM*, intramuscular.

The methodology is summarized in Figure 3. In brief, an online BWS survey was developed in which respondents were presented with 4 case vignettes (selected from a total of 32 vignettes; Table I) and asked to select the pair that, in their opinion, reflected the maximum difference in severity. Respondents were then presented with a different set of 4 vignettes, which may have included some already presented to the respondent and selected again (by the underlying BWS algorithm) in order to evaluate user preference. This process was repeated around 20 to 30 times (as determined by the BWS algorithm), with respondents asked each time to express their preference for the most and least severe reactions. This allowed the algorithm to evaluate the pattern of responses and generate a preference ranking (representing severity) for each case scenario for each respondent. Responses from all respondents were then modeled and a preference score (representing severity ranking) determined for each scenario.

**Table I:** Case vignettes used in the BWS exercise∗

**NOT Anaphylaxis**
1. Age 33 y. 5 min after eating apple, peach, and kiwi in a fruit salad: oral and throat itch. No other symptoms. Resolves spontaneously in < 30 min without medication.
2. Age 7 y. After eating some cake mix containing raw egg: perioral urticaria. No other symptoms. Resolves spontaneously within 10 min.
3. Age 16 mo. 5 min after several bites of an omelet: perioral erythema and itchy face. No other symptoms. Symptoms resolve without treatment.
4. Age 22 y. Immediately after eating shrimp: oral itch and lip edema. No other symptoms. Reaction resolves within 1 h, without treatment.
5. Age 4 y. 5 min after eating a few teaspoons of fish: oral itch and perioral erythema, then nausea and abdominal pain; 20 min later, he vomits once. Symptoms improve rapidly thereafter.
6. Age 2 y. Immediately after drinking 5 spoons of fish soup: swollen lips and perioral urticaria. Vomits 10 min later. Given an oral antihistamine, all symptoms resolve within 30 min.
**7. Age 17 y. Within minutes of eating cake containing hazelnut: itchy mouth and mild lip swelling. Over next 15 min: abdominal cramps and urticaria over the neck and legs. Develops a headache; upon standing, he feels clammy (sweaty), looks pale, and almost faints. He lies down and all symptoms resolve within 15 min.**
8. Age 6 mo. Within 5 mins of drinking 20 mL of milk formula: facial erythema and angioedema (lips, eyes). Symptoms quickly progress to generalized urticaria and she becomes irritable. No other symptoms. Given oral antihistamine, symptoms resolve within 2 h.
9. Age 42 y. Immediately after eating 3 shrimp: oropharyngeal itch and itchy red eyes, progressing to generalized pruritus with widespread skin erythema and urticaria. Chest tightness, but no cough, wheezing, or dyspnea. Symptoms resolve spontaneously without treatment.
**10. Age 18 y. 5 min after eating 2 peanuts: generalized itch and urticaria over whole body. No respiratory symptoms. On arrival at hospital, vital signs normal, chest clear. Given IM antihistamine, symptoms resolve.**
11. Age 6 mo. After drinking 50 mL of cow's milk formula: perioral urticaria and facial angioedema, which almost prevents him from opening his eyes. Given oral antihistamine and the reaction subsides within 1 h.
12. Age 18 y. 30 min after eating 3 peanuts: generalized urticaria and nausea. Normal vital signs, no respiratory compromise when assessed by a paramedic. Nausea resolves after 30 min but the urticaria persists for 4 h despite IM antihistamine.
13. Age 38 y. 30 min after eating a peach: facial angioedema, generalized urticaria, difficulty swallowing, and nausea. He attends hospital where his vital signs and respiratory examination are normal. Given IM antihistamine, symptoms resolve shortly afterward.
**14. Age 22 y. 20 min after eating some Chinese food: Abdominal cramps that persist for 30 min, until she vomits. Then develops rhinitis over the next 15 min. All symptoms resolve within 1 h.**
**15. Age 9 y, well-controlled asthma with no recent symptoms. 10 min after eating 2 peanuts: persistent cough and wheeze, which persist despite 4 puffs of albuterol via spacer. Parent administers epinephrine autoinjector, resulting in rapid resolution of reaction.**
16. Age 12 mo. 20 min after drinking 50 mL milk formula: mild facial angioedema, becomes floppy and quiet (but no loss of consciousness). BP normal. Symptoms resolve within 1 h.
17. Age 19 y. 15 min after eating a Thai curry: oral itch, facial angioedema, and severe generalized urticaria. Vital signs normal, no respiratory symptoms when assessed by a paramedic. Given IM epinephrine, symptoms resolve within 15 min.
18. Age 12 y. Within minutes of eating a chocolate: itchy mouth and slight difficulty in swallowing. After 10 min, he finds it “tight” to breathe and has a dry cough; no wheeze. Symptoms respond to 4 puffs salbutamol, but he develops mild facial erythema 20 min later.
19. Age 14 y. 10 min after eating a nut in a chocolate bar: facial angioedema, rhinitis, itchy red eyes and oral itch, and abdominal pain. Pain increases over next 45 min. She then vomits 3 times in quick succession, following which she feels much better. Widespread urticaria noted over arms and legs after vomiting.
**20. Age 24 y. Immediately after eating 2 peanuts: itchy throat, obvious rhinitis, and intermittent throat clearing/cough. Vomits 30 min later and develops a hoarse voice. All symptoms resolve without treatment after 20 min.**
**Anaphylaxis**
21. Age 18 y. Within minutes of eating a biscuit: itchy mouth and inner ear canal, progressing within minutes to itchy palms, generalized erythema, throat tightness, and vocal hoarseness. No respiratory symptoms. Normal BP recorded by paramedic, IM epinephrine given with rapid resolution of symptoms.
22. Age 24 y. Within minutes of eating some melon: oropharyngeal itch, followed by throat tightness, cough, and wheezing. Responds quickly to inhaled albuterol and IM antihistamine, given in hospital.
23. Age 8 y. 20 min after eating some cake: itchy mouth, quickly progresses to lip and tongue angioedema. Throat tightness, hoarse voice, and abdominal pain. No dyspnea. Taken to hospital, vomits once on the journey. On arrival, her voice has normalized, vital signs normal, no wheeze. Symptoms resolve over the next 1-2 h.
**24. Age 11 y. Within minutes of drinking a milkshake by accident: throat tightness, rhinitis, eyelid edema, and intermittent cough. A school nurse hears a soft inspiratory stridor and some wheeze. Little response to an initial dose of IM epinephrine, but his respiratory symptoms are relieved by nebulized albuterol and a second epinephrine dose.**
25. Age 5 y. 1 h after eating 2 shrimp: progressive generalized urticaria, red itchy eyes, throat tightness, nausea, hoarse voice, cough, and wheezing. He feels dizzy but BP is normal (assessed by paramedics). Given IM steroids, symptoms resolve within 30 min.
26. Age 11 y. Within 15 min of eating kiwi fruit: intense oral itch followed by throat tightness, with rapid progression to hoarse voice, pain on swallowing, nausea, cough, wheezing, generalized erythema/pruritus, and facial angioedema. Vomits once, 10 min later. Symptoms improve with inhaled albuterol and IM steroid.
27. Age 14 y. 10 min after eating breakfast: oral itch, throat tightness, hoarse voice, chest tightness, and persistent cough. 15 min later, she vomits, and her chest and throat feel tighter. Develops widespread itch and facial angioedema. Takes an oral antihistamine, symptoms resolve within 30 min.
28. Age 24 y. 30 min after eating pizza: facial erythema, eyelid edema and rhinitis, rapid progression to cough and wheezing. Attending paramedics note he is confused, and administer IM epinephrine with a rapid response. Given IV steroids and antihistamine *en route* to hospital, all symptoms resolve within 30 min.
29. Age 26 y. While running to work (1 h after breakfast): palmar and genital itch, generalized erythema, nausea, and abdominal pain. Vomits 5 min later and feels dizzy. Self-administers IM epinephrine. Paramedics attend, BP 70/35. Given 2 more doses of IM epinephrine and an IV fluid bolus, following which symptoms resolve.
30. Age 19 y. 15 min after eating a snack bar: chest tightness and dyspnea, feeling weak and dizzy. Taken to hospital, noted to have generalized urticaria and facial pallor. Heart rate is 110, respiratory rate 30, BP 70/40. Given nebulized albuterol, IV antihistamine, steroid, and IV fluids. Symptoms resolve within 30 min of treatment.
31. Age 15 y. 10 min after eating some chocolate: dizziness and chest tightness. Starts to cough. Paramedic reports wheezing and low BP 75/40. Symptoms resolve with 1 dose of IM epinephrine.
32. Age 32 y. 30 min after eating a curry in a restaurant: itchy palms/feet. He then faints, and is incontinent of feces. Recovers consciousness within minutes, noted to have generalized erythema and lip angioedema. Taken to hospital and given IV antihistamine and steroid. All symptoms resolve within 2 h.

*BP,* Blood pressure; *IM,* intramuscular; *IV,* intravenous.

∗ In 6 vignettes, the BWS ranking differed significantly from the expected ranking; these are highlighted in bold.

**Figure E1:**
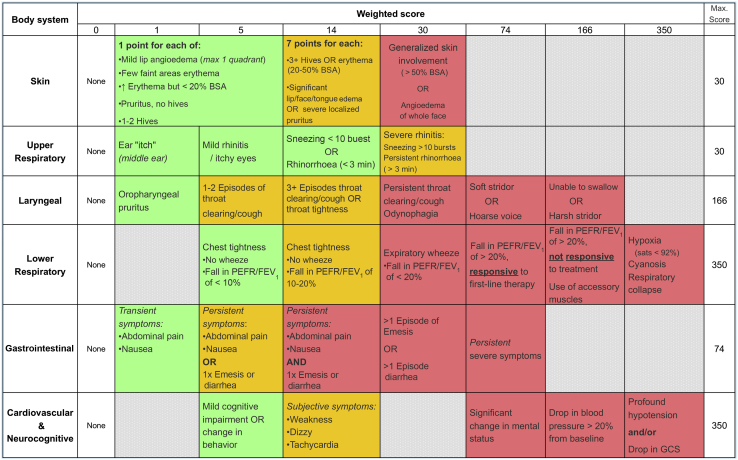
Pilot quantitative severity score developed for food-induced allergic reactions. *BSA,* Body surface area; *GCS,* Glasgow Coma Scale; *PEFR/FEV*_*1*_*,* Peak expiratory flow rate/forced expiratory volume in 1 second.

**Figure E2:**
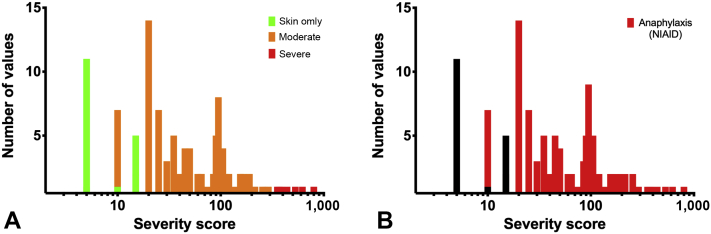
Distribution of severity scores from food-induced reactions in the EDA cohort,^e2^ according to (**A**) Brown's grading system and (**B**) NIAID anaphylaxis. EDA, emergency department anaphylaxis.

The 32 case vignettes were chosen from a bank of over 100 real-world reaction vignettes that were then reduced to provide a spectrum of reaction severity using focus groups of clinicians and a pilot survey. In order to obtain a balance encompassing a spectrum of severity of allergic symptoms, scenarios chosen included 12 reactions that were nonanaphylaxis by any definition, 12 reactions that were consistent with anaphylaxis (National Institute of Allergy and Infectious Disease/Food Allergy and Anaphylaxis Network [NIAID/FAAN] definition), and 8 reactions in which the anaphylaxis classification was more ambiguous. A quantitative score was developed to facilitate the selection of scenarios from a bank of real-life reactions (Figures E1 and E2; available in this article's Online Repository at www.jaci-inpractice.org). In pilot work, we found that displaying 5 scenarios at any one time took longer for respondents to process and was more difficult to assess than using 4 scenarios—even though this required each respondent to view more screens (sets of 4 scenarios) overall. The final survey, therefore, presented respondents with 4 scenarios per screen.

The surveys were administered by ResearchNow. Potential respondents were contacted through the Australasian Society of Clinical Immunology and Allergy (ASCIA), British Society for Allergy and Clinical Immunology (BSACI), the Spanish Society of Allergy and Clinical Immunology (SEIAC), the Food Allergy Interest Group of the European Academy of Allergy and Clinical Immunology (EAACI), and the World Allergy Organization (WAO). Responses were voluntary and confidential. Ethical approval was not required for this research project; the study was approved by the Executive Committee of the WAO and the U.K. Market Research Society's Code of Conduct was followed.

### Statistical considerations

There are no formal methods to determine sample size for BWS analyses.^21^ The required sample size is dependent on the number of scenarios included in the study and the number of case vignettes shown at any one time. ResearchNow advised that a potential response of 250 to 300 healthcare professionals would allow a total number of 32 scenarios to be used. Given differences in the clinical criteria for anaphylaxis globally^1^ (and possible different thresholds for recommending epinephrine treatment), we evaluated differences in BWS ranking between respondents in North America and in Europe. We also assessed the impact of experience (years of practice, exposure to positive food challenges) on responses. These analyses were done using the unpaired *t* test. In line with established methods for BWS analyses, statistical significance was set at *P* less than .05, with no correction for multiple comparisons.

Each of the 32 scenarios included the BWS exercise were scored independently by 3 clinicians (A.S., P.J.T., M.F.-R.), using other published severity grading systems.^5^, ^6^, ^7^, ^8^, ^9^, ^10^, ^11^, ^12^, ^13^, ^14^, ^15^, ^16^, ^17^^,^^19^ Discrepancies were resolved through discussion and consensus. For each grading system, the severity ranking of the scenarios (determined by the grading system) was compared with the ranking derived by the BWS exercise, using Spearman r correlation and weighted Cohen kappa level of agreement. For the latter, we categorized the BWS ranking into a number of categories equivalent to that used by the comparator grading system (ie, for scoring systems with 5 grades, the BWS rankings were mapped into quintiles). Cohen kappa is limited by the need to compare categories rather than continuous measures. We, therefore, also undertook a comparison of area under the curve for the resulting severity plots with the equivalent plot for the BWS ranking and used this to assess whether each scoring system over- or underestimated severity compared with the BWS ranking.

## Results

### Characteristics of respondents

Complete responses were received from 334 health care professionals spanning 45 countries: 183 (55%) were from Europe, 65 (20%) from North America, and 86 from the rest of the world (Table E1; available in this article's Online Repository at www.jaci-inpractice.org). Owing to overlap in the membership lists between ASCIA, BSACI, EAACI, SEAIC, and WAO and data protection issues, we were unable to calculate a response rate. A total of 257 respondents (77%) had a medical qualification; the majority were specialist physicians in allergy (66%), whereas 29 (9%) were general pediatricians and 28 (8%) specialist allergy nurse practitioners (Table E2; available in this article's Online Repository at www.jaci-inpractice.org). A total of 122 (37%) respondents predominantly saw pediatric patients only, 96 (29%) saw adults, and the remainder both. A total of 204 (61%) had been in clinical practice for over 10 years; 44% of all respondents reported witnessing positive oral food challenges on a regular basis (Table E3; available in this article's Online Repository at www.jaci-inpractice.org).

### BWS ranking score as assigned by respondents

**Figure 4:**
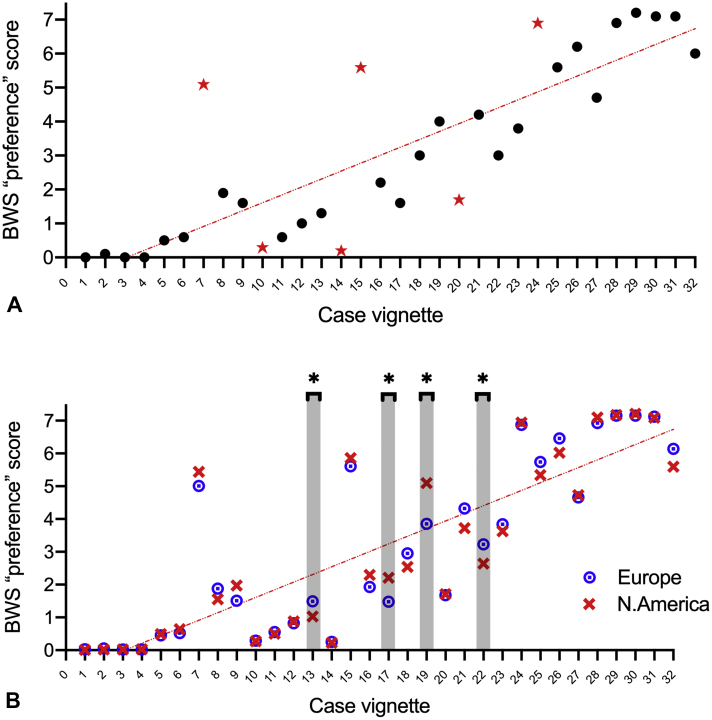
(**A**) BWS preference score for each case vignette, ranked according to the pilot quantitative severity score (see Online Repository at www.jaci-inpractice.org)). Six potential outliers are highlighted by red stars. (**B**) BWS preference score by location of respondent (Europe vs North America). ∗*P* < .05.

Figure 4A shows the BWS preference score for each scenario, ordered according to the quantitative score developed to facilitate this exercise (described in the Online Repository; available in this article's Online Repository at www.jaci-inpractice.org). There were 6 case vignettes in which the BWS ranking differed significantly from the expected ranking, as highlighted in Table I. Analyzing the BWS ranking according to geographical location of respondents (Figure 4B), those based in North America rated cases 17 and 19 as being of a higher severity, compared with European respondents (*P* < .05). Case 17 described a reaction involving mucocutaneous symptoms only, treated with epinephrine. Case 19 is a scenario that arguably might be described as anaphylaxis according to NIAID/FAAN criteria,^23^ but not viewed as anaphylaxis in the United Kingdom and Australia.^24^ Cases 13 and 22 were rated as lower severity by North American respondents than by those in Europe; both these cases described reactions to fruits involving lipid transfer protein allergy.

**Figure 5:**
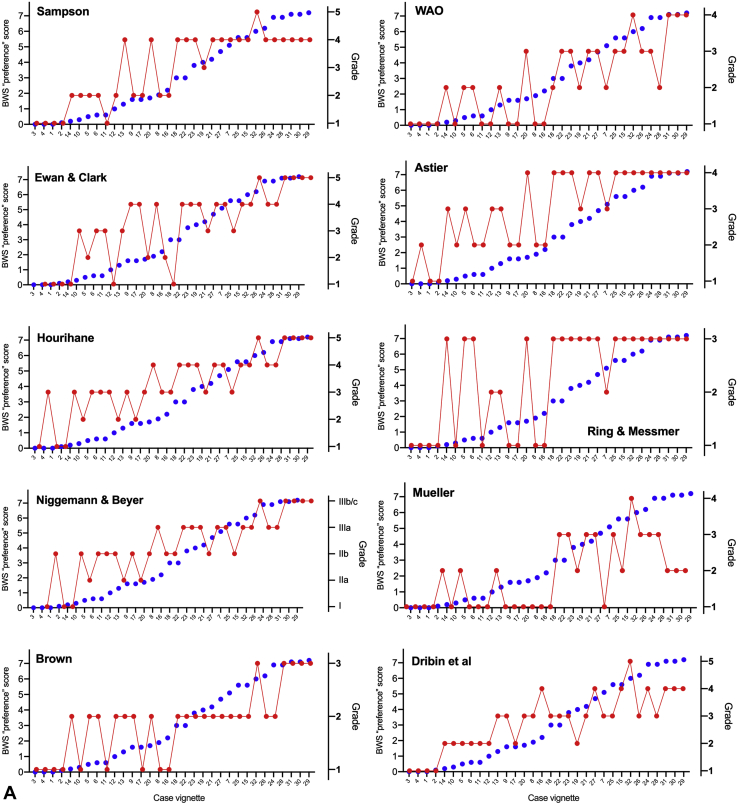

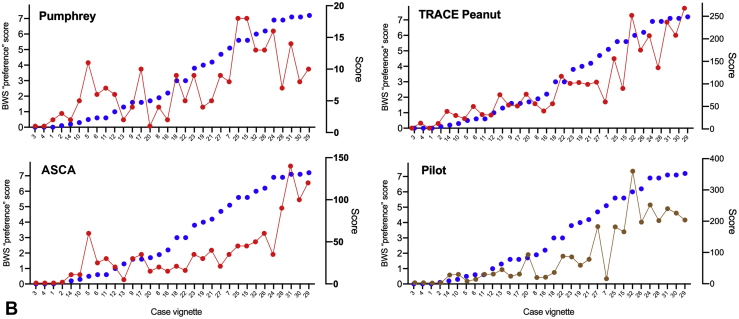
Comparison of BWS ranking (in blue) with existing severity scoring systems used to grade food-allergic reactions.

**Figure E3:**
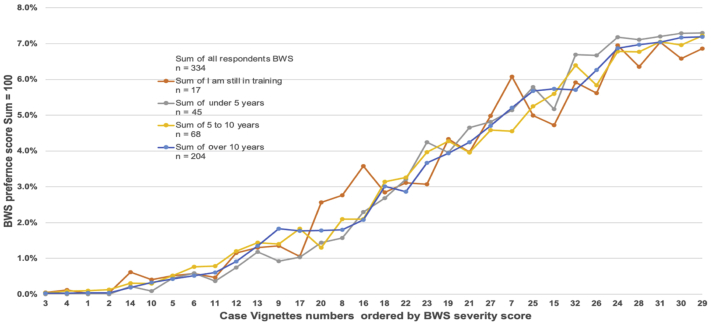
BWS preference score by years of experience.

**Figure E4:**
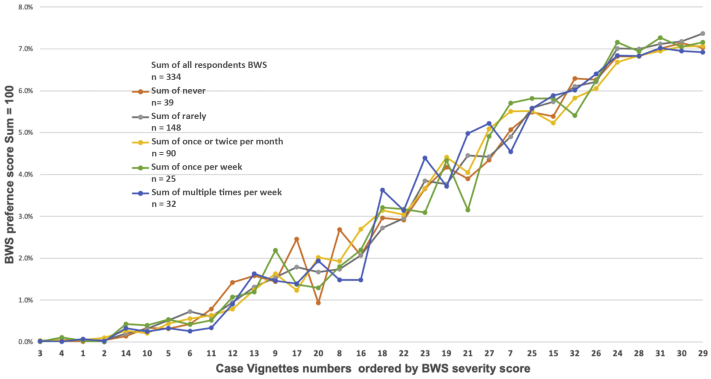
BWS preference score by exposure to positive food challenges.

Comparing specific scenarios with one another also provided some insight into the factors that might impact on severity perception. For example, case 17 describes a widespread cutaneous reaction that was treated with self-administered epinephrine. This reaction was rated significantly higher (*P* < .05, *t* test) by respondents than case 10 (almost identical but without epinephrine being used), a trend that was most marked for those with less than 10 years' experience. Case 17 was also rated as more severe than case 13, which was similar in terms of cutaneous symptoms but also described dysphagia and nausea. This suggests that the use of epinephrine, even for non-anaphylaxis reactions, subconsciously sways the perception toward one of greater severity, particularly in those with less experience. Respondents still in training tended to assign certain vignettes as being of greater severity than did more senior clinicians (Figure E3; available in this article's Online Repository at www.jaci-inpractice.org). Case vignette 7, a scenario in which a patient with a systemic but non-anaphylaxis reaction almost fainted on standing, was rated as less severe by more senior respondents. We also evaluated the impact of regular exposure to positive challenges on severity assignment (Figure E4; available in this article's Online Repository at www.jaci-inpractice.org). Those with greater exposure ranked scenarios with laryngeal symptoms (eg, vocal hoarseness) as more severe than those with less regular exposure to positive food challenges (case vignettes 20, 21, and 27).

### Comparison of BWS preference score versus existing severity scores

**Figure E5:**
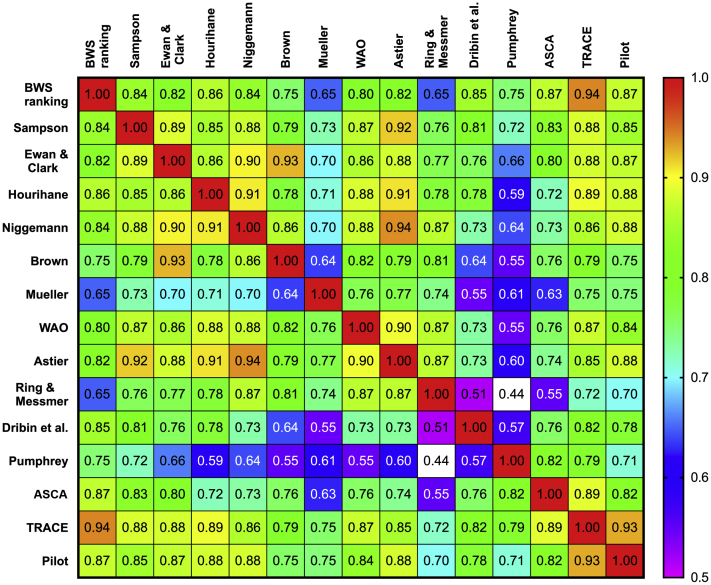
Spearman r_s_ coefficients for the different severity scores included.

Figure 5 shows the score for each case vignette using existing grading systems described in the literature compared with the BWS ranking. For these comparisons, cases were ordered in terms of the BWS ranking. The level of agreement with the BWS ranking, together with degree of correlation, is reported in Table II. A full analysis of correlation between the different scores can be found in Figure E5 (available in this article's Online Repository at www.jaci-inpractice.org).

**Table II:** Comparison of different symptom severity systems to the BWS ranking, using Spearman r_s_ and agreement assessed by Cohen kappa and using AUC (see Methods)∗

Severity score	Correlation to BWS rank (Spearman r_s_)	Agreement (Cohen kappa)	Agreement (AUC)	Comments
Sampson^5^	0.84	0.63 substantial	Overall: 117% • Mild: 330% • Moderate: 141% • Severe: 88%	Overestimates severity, especially for mild reactions. May be due to • Pharyngeal symptoms and low-grade tachycardia assigned a greater relative severity (ie, grade 3) • Loss of consciousness graded same as cardiac or respiratory arrest
Ewan & Clark^6^	0.82	0.59 moderate	Overall: 131% • Mild: 508% • Moderate: 141% • Severe: 97%	Overestimates severity, especially for more mild reactions. Better agreement for more severe reactions. Overestimate may be due to • Even mild ear/nose symptoms being assigned as grade 3 severity • Throat tightness assigned as grade 4
Hourihane et al^7^	0.86	0.48 moderate	Overall: 138% • Mild: 620% • Moderate: 160% • Severe: 97%	Overestimates severity, especially for more mild reactions. Better agreement for more severe reactions. Overestimate may be due to non-urticaria symptoms being graded as grade 3+, leading to mild reactions that include non-skin symptoms being assigned a higher grade of severity.
Niggemann & Beyer^8^	0.84	0.59 moderate	Overall: 125% • Mild: 531% • Moderate: 135% • Severe: 93%	Overestimates severity, especially for mild reactions. Fairly good agreement for significant reactions and anaphylaxis but tendency to overrate mild reactions because skin + GI symptoms propels into mid-range grade, resulting in poor discrimination of non-anaphylaxis reactions.
Brown^9^	0.75	0.56 moderate	Overall: 108% • Mild: 489% • Moderate: 97% • Severe: 86%	Despite limited discrimination with only 3 grades, the grading system performs very well compared with other scores. Tendency to overrate non-anaphylaxis reactions because any non-cutaneous symptoms—however minimal—propels into mid-range severity.
Mueller^10^	0.65	0.46 moderate	Overall: 73% • Mild: 222% • Moderate: 75% • Severe: 63%	Tendency to underrate more severe reactions because higher grades need 2 or more severe symptoms (rather than just 1 severe symptom alone).
WAO^11^	0.80	0.53 moderate	Overall: 97% • Mild: 280% • Moderate: 99% • Severe: 85%	Only grade 1 reactions constitute non-anaphylaxis, resulting in a lack of discrimination for non-anaphylaxis reactions. Most discriminatory for wheeze (2 possible grades), with good agreement with BWS ranking. Laryngeal symptoms are ignored by the WAO score.
Astier et al^12^	0.82	0.52 moderate	Overall: 161% • Mild: 716% • Moderate: 188% • Severe: 111%	Overestimates severity, especially for mild-moderate reactions. Score is based on the number of organs involved, symptoms involving 2 or more organs are assigned grade 3, even if symptoms are mild. 3+ organs are reassigned a minimum of grade 4 severity, overestimating severity.
Ring & Messmer^13^	0.65	0.45 moderate	Overall: 156% • Mild: 778% • Moderate: 171% • Severe: 110%	Poor agreement with significant overestimation of severity of food reactions • Vomiting/laryngeal symptoms ranked as grade 3, overestimating severity • Bronchospasm is equivalent to cyanosis, lacks discrimination for wheeze • No discrimination for skin symptoms (severe skin symptoms only grade 1)
Dribin et al^17^	0.85	0.50 moderate	Overall: 132% • Mild: 352% • Moderate: 126% • Severe: 77%	Overestimates severity for mild-moderate reactions, underestimates severity for more severe reactions. Mild subjective cardiovascular, respiratory, or neurological symptoms given more prominence than moderate-severe GI or moderate cutaneous symptoms.
Pumphrey and Stanworth^15^	0.75	NA as continuous (rather than ordinal) score	Overall: 89% • Mild: 393% • Moderate: 71% • Severe: 79%	Does not score for nausea or abdominal pain (which are common in reactions due to food). Tendency to underestimate severity, perhaps because • Recurrent vomits/uterine cramps ranked as grade 2 only • Low-grade throat symptoms score highly
ASCA^16^	0.87	NA as continuous (rather than ordinal) score	Overall: 59% • Mild: 214% • Moderate: 45% • Severe: 57%	Underestimates non-mild symptoms: the ASCA scoring system assigns a higher weighting to mild-moderate cardiovascular symptoms (eg, tachycardia) than to respiratory symptoms (eg, wheeze).
TRACE Peanut^18^^,^^19^	0.94	NA as continuous (rather than ordinal) score	Overall: 79% • Mild: 184% • Moderate: 73% • Severe: 76%	Similar to other scoring systems, this overestimates mild symptoms but underestimates more severe symptoms.

*AUC,* Area under the curve; *GI,* gastrointestinal; *NA,* not applicable.

∗ For the AUC, <100% is an underestimate of severity, whereas a figure > 100% indicates an overestimate of severity. Figures are given for each tertile, corresponding to mild, moderate, and more severe reactions.

## Discussion

Using a novel approach based on BWS methodology and informed by 334 respondents (all members of specialist allergy societies), we defined a scenario-based severity ranking that we then compared with existing severity scoring systems for food allergy in the literature. Our analysis demonstrated a number of limitations with the existing scoring systems. Some were relevant across the board, namely, a lack of discrimination between non-anaphylaxis symptoms of differing severity. Other limitations (as highlighted in Table II) identified organ-specific issues, in which one scoring system might assign a greater or lower level of significance to a set of symptoms, resulting in a lack of agreement with the BWS ranking (and each other). Currently, there are multiple and often inconsistent approaches in assigning severity of an allergic reaction, many of which were explored in this study.

Previous attempts to develop severity scores have essentially been informed by a very limited number of experts. Dribin et al^17^ recently proposed a new score developed through Delphi consensus, but only 21 experts were involved, which still risks a high degree of subjectivity. A more preferable approach might be to use objective data to derive—or at least inform—a score.^20^ However, this would still require some form of validation to a gold standard. This BWS exercise provides a less biased consensus framework informed by over 300 allergy health care professionals, which could potentially be applied to the development of a comprehensive validation tool against which existing and future severity scores can be compared. To our knowledge, the only other comparison of severity scores in the literature is a comparison by Eller et al,^25^ who retrospectively applied 23 different scoring systems to 2,848 reaction reports from a single medical center. The authors found that there was often poor agreement as to severity between different scores and that a scoring system for one allergen trigger (eg, medication) could not be easily translated to other triggers (such as food)—particularly those designed for hymenoptera venom–triggered reactions.

The initial selection of cases vignettes was informed by a pilot quantitative severity score (described in the Online Repository; available in this article's Online Repository at www.jaci-inpractice.org). The BWS exercise identified 6 scenarios that were assigned a very different level of severity by the BWS preference score compared with the pilot score. Case vignette 7 described a reaction involving vasovagal syncope; respondents may have interpreted this scenario as cardiovascular compromise with hypotension due to anaphylaxis, hence assigning a higher severity ranking. However, we recently published data suggesting that vasovagal instability, resulting in orthostatic intolerance, can occur in up to 11% of participants reacting to peanut at a supervised food challenge.^26^ This suggests a lack of awareness that such symptoms can occur during food-induced allergic reactions and might not suggest anaphylaxis (in terms of cardiovascular compromise due to distributive shock).

Cases 10 (isolated but generalized cutaneous symptoms) and 14 (initially, isolated gastrointestinal symptoms [of 30 minutes' duration] relieved by vomiting that caused rhinitis) were ranked of lower severity than expected. Case 15 (a patient with persistent cough and wheeze following peanut ingestion) was assigned a higher severity ranking than expected; this may be due to the lack of response to salbutamol/albuterol, a scenario that is not uncommon given the transient impact of bronchodilators on respiratory symptoms in the context of anaphylaxis. Case 20 described a reaction with laryngeal involvement; this scenario was assigned a lower BWS ranking than expected, possibly owing to laryngeal symptoms not being considered as indicating severity (given the absence of laryngeal symptoms in the NIAID/FAAN anaphylaxis definition)^17^ and/or the fact that symptoms resolved without treatment. Case 24 was a milk-induced reaction with cutaneous, upper and lower respiratory involvement; this may have been assigned a higher severity score because treatment involved 2 doses of epinephrine. Dribin et al recently undertook a Delphi exercise with an expert panel to define a new severity score and specifically excluded treatment response on the basis that “use of these therapies may be subject to medication availability and practice variation.”^17^ Such an assertion is supported by data from the PALISADE^27^ and ARTEMIS^28^ studies, which indicated a higher rate of epinephrine use at baseline peanut challenges in North America than in Europe despite very similar study protocols. If treatment is reflective of severity alone, then one could erroneously conclude that peanut-allergic individuals in North America have more severe reactions than their European counterparts. Whether treatment should inform severity assignment remains controversial.^20^ Given that epinephrine is often used for non-anaphylaxis reactions, although many anaphylaxis reactions are not treated with epinephrine in the community, severity should perhaps be considered independent of treatment. However, an anaphylaxis reaction that is refractory to epinephrine treatment is arguably more severe than one that responds to 1-2 doses of epinephrine. This is presumably why the WAO grading system for reactions to subcutaneous immunotherapy includes treatment response as a factor.^11^ The responses from the BWS exercise suggest that, in practice, clinicians do consider the response to treatment as a factor in determining severity, something that is currently only incorporated into one severity grading system (WAO).^11^

The BWS is a research technique to measure robust ranking of items. It is often used in market research to assess consumer preference. The approach eliminates user scale bias that can occur owing to the use of a monadic rating scale, in which respondents are asked to rate an item in isolation, without reference to other items (Figure 2). The underlying algorithms ensure a balanced presentation of items (in this study, reaction vignettes), with a preference toward obtaining more discriminatory detail for those items where the difference in severity was less clear. The design also reduces the impact of respondent fatigue, whereby responses are logged within due consideration: the algorithm can identify discordant responses and prompt further comparisons from those who have provided incongruous answers. However, there are some limitations. The BWS measures relative rather than absolute preferences.^22^ The comparison between items are dependent on the details within each scenario, but including too much detail risks user fatigue—therefore, there is a trade-off between the two.

We observed some differences in severity assignment for some scenarios, depending on the location of the respondent. In some scenarios, the ranking was higher by respondents based in North America than in Europe. This may have been due to a greater familiarity and usage of the NIAID/FAAN criteria for anaphylaxis (which includes reactions with cutaneous and gastrointestinal manifestations, which would not necessarily be classified as anaphylaxis in Europe).^24^ The relative prevalence of different food triggers might have also influenced the perception of severity. Three scenarios described food-induced reactions to fruits (cases 13, 22, and 26), at least 2 of which were likely to be due to lipid transfer protein allergy,^29^ a phenomenon that is perhaps more familiar to many European clinicians (particularly those from the Mediterranean region) than respondents located in North America.

Previous experience, in terms of both seniority (years of independent practice) and familiarity with positive food challenges, tended to impact on the perception of severity of respondents. Respondents still in training or less familiar with positive food challenges did not always recognize subtle symptoms that more experienced clinicians attributed a higher degree of severity to, for instance, vocal hoarseness and itchy palms. Experience is not a straightforward concept. It is not unusual for more-experienced clinicians to work more remotely from acute patient visits, with the consequence that the most senior clinicians might be those who are less exposed to positive food challenges on an ongoing basis. As a result, we used both years post qualification as well as direct exposure to positive food challenges to define experience. Although not statistically significant, we observed a trend in which respondents—often those with less experience—tended to assign a higher severity rating in the BWS exercise if epinephrine was administered, even if the scenario did not describe a reaction consistent with anaphylaxis by any definition. Epinephrine use is not necessarily a reliable marker of severity: anaphylaxis reactions are frequently undertreated with epinephrine,^1^, ^2^, ^3^^,^^23^ despite this being the first-line intervention for anaphylaxis in all international guidelines.

This study is not without limitations. Although we achieved the minimum 300 responses, clinicians who participated in the BWS exercise still represent a relatively small proportion of healthcare workers practicing in clinical allergy. Ideally, we would repeat this study with more participants from underrepresented regions as well as from other disciplines (such as emergency medicine), so we can compare different regions and ascertain where they may be influenced by different definitions, levels of education, and training. Existing severity scores depend on the presence or absence of specific symptoms, although at least one also includes treatment response.^11^ In reality, the perception of severity is also dependent on other factors, such as timing of onset of symptoms, spontaneous resolution, and need for and subsequent response to treatment. The BWS exercise reflected this reality, but undertaking the exercise was not a quick task, especially for respondents for whom English was not their first language. It typically took 40 to 60 minutes to complete, which increased the risk of response fatigue, although the underlying algorithms did not detect this as an issue in the responses received. Ideally, the survey should have been translated into multiple languages, but owing to significant cost implications, this was not feasible. Ideally, we might have used videos of reactions rather than case vignettes to overcome some limitations including language; however, obtaining video recordings of real-world reactions, particularly occurring outside a healthcare environment, would be extremely challenging.

The management of food allergy involves multiple stakeholders, often with differing perspectives regarding severity. Further work could adapt the premise of this exercise to assess how other stakeholders (including patients themselves) perceive reactions and how this differs from healthcare professionals. This methodology could also be expanded to assess severity scoring assignment of different allergen triggers beyond just foods. Understanding factors that influence how patients rank severity could assist us in how we counsel patients and encourage the more appropriate treatment of reactions. A better understanding of how response to treatment impacts on perception of severity would be useful in clinical practice—an approach that could potentially save lives if this facilitated the development of tools to help identify reactions refractory to epinephrine and prompt rapid escalation of treatment. Incorporating response to treatment as an indicator of severity could help prompt the need to escalate intervention and guide further treatment.

## Summary

Through a global BWS exercise, we identified a number of significant limitations (summarized in Table III) with existing severity scores used to grade food-induced allergic reactions, which varied from one score to another. Having a standardized, internationally agreed-upon quantitative measure for severity would be useful for the accurate assessment and improved communication of reaction severity, which could be applied to healthcare as well as influencing food businesses and regulatory bodies. Increasing our ability to accurately and consistently assign severity should lead to improvements in the management of patients with food allergy. These data provide a potential method, free of user scale bias, toward defining a consensus-driven gold standard that can be used to guide and validate future severity scores.

**Table III:** Summary of current limitations with existing severity scores

• **Poor discrimination between nonanaphylaxis reactions of differing severity,** which results in an apparent overestimation of severity by current scores (because even very mild symptoms are rated as severe as generalized but non-anaphylaxis cutaneous reactions)
• **Lack of transferability of a score designed for nonfood triggers to food-induced reactions,** for example, owing to emesis being graded as a very severe symptom by some systems (particularly those for venom allergy), which may not be the case for food-induced reactions
• **Anaphylaxis is a poor determinant of severity,** eg, severe generalized cutaneous reactions may be more severe than mild anaphylaxis reactions
• **Distinguishing between use of a particular treatment, and the *response* to that treatment:** for example, epinephrine use is a poor marker of severity. However, a lack of treatment *response* (as opposed to treatment itself) does seem to be important in assigning severity.
• **Assignment of “soft” symptoms** such as vasovagal syncope or laryngeal symptoms, which are not captured by many current grading systems.

## Figures and Tables

**Table E1:** Respondents by country of origin

Country	Respondents (n)
Europe • Austria • Belgium • Croatia • Czech Republic • Denmark • Finland • France • Germany • Greece • Hungary • Ireland • Italy • Netherlands • Poland • Portugal • Romania • Spain • Sweden • Switzerland • UK	183 2 3 4 2 2 3 4 5 3 3 4 14 10 4 9 8 39 3 3 49
North America • United States • Canada • Mexico	65 47 8 10
Rest of world • Argentina • Australia • Brazil • Chile • China • India • Israel • Malaysia • Russia • Singapore • Thailand • Turkey	86 4 16 10 2 2 4 3 4 3 3 2 8

**Table E2:** Respondents listed by specialty

Role in the management of patients with food allergy	Respondents, n (%)
Allergist	221 (66)
CNS allergy	28 (8)
Pediatrician	29 (9)
Family practitioner	7 (2)
Dietitian	14 (4)
Other	35 (10)

*CNS,* Central nervous system.

**Table E3:** Respondents listed by level of experience

Proportion of pediatric patients seen in clinic (%)	Respondents, n (%)
0-25	96 (29)
25-50	74 (22)
51-75	42 (13)
76-95	25 (7)
96-100	97 (29)
Years qualified in role (y)	Respondents, n (%)
Still in training	17 (5)
<5	45 (13)
5-10	68 (20)
>10	204 (61)
Frequency of direct observation of positive food challenges	Respondents, n (%)
Never	39 (12)
Rarely	148 (44)
Once or twice per mo	90 (27)
Once per wk	25 (7)
Multiple times per wk	32 (10)
Never	39 (12)

## Supplementary methods

The 32 case vignettes were chosen from a bank of over 100 true reaction descriptions. In order to obtain a balance encompassing a spectrum of severity of allergic symptoms, we developed a quantitative severity score, based on the weighting of symptoms categorized according to the PRACTALL consensus,^e1^ by modifying and extending a symptom score first proposed by Lockey et al.^14^ This symptom score is shown in Figure E4. This was further refined and tested using symptom and biomarker data from a large cohort of patients with anaphylaxis (n = 131) presenting to emergency departments in Australia.^e2^ These data, evaluated by 2 independent clinicians (P.J.T., A.A.) using the quantitative severity score fitted a log-normal distribution (Shapiro Wilk normality test) with good discrimination between reactions of differing severity (Figure E5).
